# *In silico* Approaches for the Design and Optimization of Interfering Peptides Against Protein–Protein Interactions

**DOI:** 10.3389/fmolb.2021.669431

**Published:** 2021-04-28

**Authors:** Zahra Sadat Hashemi, Mahboubeh Zarei, Mohsen Karami Fath, Mahmoud Ganji, Mahboube Shahrabi Farahani, Fatemeh Afsharnouri, Navid Pourzardosht, Bahman Khalesi, Abolfazl Jahangiri, Mohammad Reza Rahbar, Saeed Khalili

**Affiliations:** ^1^ATMP Department, Breast Cancer Research Center, Motamed Cancer Institute, Academic Center for Education, Culture and Research, Tehran, Iran; ^2^Pharmaceutical Sciences Research Center, Shiraz University of Medical Sciences, Shiraz, Iran; ^3^Department of Cellular and Molecular Biology, Faculty of Biological Sciences, Kharazmi University, Tehran, Iran; ^4^Department of Medical Biotechnology, Faculty of Medical Sciences, Tarbiat Modares University, Tehran, Iran; ^5^Cellular and Molecular Research Center, Faculty of Medicine, Guilan University of Medical Sciences, Rasht, Iran; ^6^Department of Biochemistry, Guilan University of Medical Sciences, Rasht, Iran; ^7^Department of Research and Production of Poultry Viral Vaccine, Razi Vaccine and Serum Research Institute, Agricultural Research Education and Extension Organization, Karaj, Iran; ^8^Applied Microbiology Research Center, Systems Biology and Poisonings Institute, Baqiyatallah University of Medical Sciences, Tehran, Iran; ^9^Pharmaceutical Sciences Research Center, Shiraz University of Medical Sciences, Shiraz, Iran; ^10^Department of Biology Sciences, Shahid Rajaee Teacher Training University, Tehran, Iran

**Keywords:** peptide, protein–protein interactions, *in silico*, bioinformatics 3, interfering peptides

## Abstract

Large contact surfaces of protein–protein interactions (PPIs) remain to be an ongoing issue in the discovery and design of small molecule modulators. Peptides are intrinsically capable of exploring larger surfaces, stable, and bioavailable, and therefore bear a high therapeutic value in the treatment of various diseases, including cancer, infectious diseases, and neurodegenerative diseases. Given these promising properties, a long way has been covered in the field of targeting PPIs *via* peptide design strategies. *In silico* tools have recently become an inevitable approach for the design and optimization of these interfering peptides. Various algorithms have been developed to scrutinize the PPI interfaces. Moreover, different databases and software tools have been created to predict the peptide structures and their interactions with target protein complexes. High-throughput screening of large peptide libraries against PPIs; “hotspot” identification; structure-based and off-structure approaches of peptide design; 3D peptide modeling; peptide optimization strategies like cyclization; and peptide binding energy evaluation are among the capabilities of *in silico* tools. In the present study, the most recent advances in the field of *in silico* approaches for the design of interfering peptides against PPIs will be reviewed. The future perspective of the field and its advantages and limitations will also be pinpointed.

## Introduction

The survival of a cell naturally depends on the connections between its proteins. In a single organism, countless cells are connected to form an interactome. The interactome is an enormous network system composed of a whole set of molecular interactions, particularly protein–protein interactions (PPIs). These interactions could be held together *via* electrostatic forces, hydrogen bonding, and hydrophobic effect. Approximately 130,000–600,000 PPIs are correlated with the human interactome and make up the PPI networks (Bruzzoni-Giovanelli et al., 2018). These PPIs modulate the systematic function of cells and signaling pathways of the body and are essential to understanding the medicinal chemistry and chemical biology of the cells. They catalyze the critical cellular processes such as replication, transcription, translation, and transmembrane signal transduction (Lu et al., 2020). Proteins and consequently PPIs are the functional building blocks of a living cell. The slightest error in PPIs, especially in a central node (or hub) in a network, could lead to even fatal disease (such as infectious diseases, cancer, and neurodegenerative diseases) and disturb the cell homeostasis. Therefore, focusing on aberrant PPIs holds the promise of curing various diseases and has the therapeutic potential of being attractive targets for developing new drugs and novel diagnostics.

Deliniation of the interaction between a protein domain and a peptide or another protein domain, is the central concept of PPIs (Nevola and Giralt, 2015). A linear sequence of residues, or a short protein domain, evokes the concept of peptides and peptide mimetics (Stone and Deber, 2017). Recently, various drugs have been presented that are relying on the relationship between two proteins. These drugs could mimic the 3D shape of targeted proteins and can specifically fine-tune their interactions. The peptides are capable of adapting secondary structures, which are usually α-helixes, which could also be completely disordered (Nevola and Giralt, 2015).

To provide the desired peptide or small molecule drug, clinical- and molecular-level information is needed (Bakail and Ochsenbein, 2016). Bioinformaticians widely apply high-throughput data obtained from the studies of genomics, RNAomics, proteomics, metabolomics, and glycomics. These collected data are rich sources of the molecular-level information of PPIs and could be useful for personalized treatments (Bakail and Ochsenbein, 2016). *In silico* methods have been widely used in various aspects of biological studies (Khalili et al., 2017; Mard-Soltani et al., 2018; Khodashenas et al., 2019; Rahbar et al., 2019). *In silico* methods could be used to screen for the specific topological surface of peptides capable of specific modulation of PPIs. There is a wide variety of software and algorithms for scrutinizing PPIs to design and optimize these interfering peptides.

Basic *in silico* research about the PPIs (from the 1990s to the 2000s) has significantly contributed to the production of a vast number of constrained peptides and peptide–drug conjugates. The first approvals of the peptide therapeutics were issued for six peptides in 2012. Pasireotide is a somatostatin analog for the treatment of Cushing’s disease. Lucinactant is a pulmonary surfactant for the treatment of infant respiratory distress syndrome. Peginesatide is an erythropoietin analog for the treatment of anemia associated with chronic kidney disease (CKD). Carfilzomib is an epoxomicin analog (proteasome inhibitor) that is used as an anticancer medication. Linaclotide is an oligo-peptide agonist of guanylate cyclase 2C used to treat irritable bowel syndrome with constipation and chronic constipation with no known causes. Teduglutide is a 33-membered polypeptide and glucagon-like peptide-2 (GLP-2) analog for the treatment of short bowel syndrome. A new field has opened up for scientists to treat different diseases with the help of various *in silico* algorithms, tools, and software designs by discovering therapeutic peptides and small molecules (Kaspar and Reichert, 2013). The results of *in silico* studies should be confirmed *in vitro* and *in vivo* to pass the phases of clinical trials. The PPI networks would be disturbed and therefore restrained the usage of these therapeutic PPI-targeting peptides (Kaspar and Reichert, 2013).

*In silico* methods of peptide analyses could include different approaches such as homology modeling, molecular dynamics, protein docking, and PPI targeting. Structural characterization of the peptides could be carried out by x-ray crystallography, NMR spectroscopy, and cryo-electron microscopy. The obtained structural data are stored and available in structural deposition databases like the Protein Data Bank (PDB). The advantages of computational *in silico* methods over empirical methods are their low cost, faster procedure speed, simple process, and reliability to target PPIs using peptides. This approach can lead to the atomic-level identification of PPIs (Murakami et al., 2017). The information about the PPIs is crucial for designing desired peptides or small molecules *via* virtual screening of drug candidates (Nevola and Giralt, 2015). Peptides are reported to have some advantages over small molecule drugs. Small molecules are not sufficient for the complete coverage of the large contact surfaces involved in PPIs. This surface area could practically be from 1,500 to 3,000 Å (Cunningham et al., 2017). Using small molecules, some target sites of the PPI surfaces such as pockets, grooves, or clefts could be missed. Advanced and sophisticated design and modeling of new remedial small molecules will be needed to circumvent this drawback. However, interfering peptides (IPs), natural or synthetic, are superior in this respect and would be reconciled with large or flat PPI surfaces (Bruzzoni-Giovanelli et al., 2018). The studies have shown that the ADME properties of IP therapeutic agents are better than those of small molecules. ADME is correlated with the absorption, distribution, metabolism, excretion, and toxicity of a drug molecule. IPs could be simply designed as valuable therapeutic tools to block various PPI networks. Peptides have been analyzed to block PPIs of the nervous system (Zhai et al., 2014; Shi et al., 2016), cardiovascular system (Pleasant-Jenkins et al., 2017), and even cancer (Jouaux et al., 2008; Mine et al., 2016; Ellert-Miklaszewska et al., 2017). IPs are also associated with low molecular weight, high flexibility, and minimal toxicity, which could offer a new class of biopharmaceuticals. These small peptides could also act as cargo carriers due to their cell penetration property. These cargoes could be linked *via* covalent or non-covalent bonds to some short peptides (5–30 amino acids). These peptides, which could have positive charges, are called cell-penetrating peptides (CPPs). CPPs facilitate cellular delivery and uptake of their cargo commonly through endocytosis (Guidotti et al., 2017). The prospect of IP drugs shows a robust pipeline and an unrivaled number of marketing opportunities for the treatment of a wide range of diseases.

## Databases for Peptide Sequences and Structures

Peptides, once neglected, have now offered tremendous therapeutic applications (Antosova et al., 2009) and have obtained quite an expansion in the pharmaceutical industry. Numerous databases have already been developed to store different kinds of peptides by collecting information from public databases and published scientific articles (Table 1). These repositories can be categorized into three classes, namely antimicrobial peptide (AMP) databases, targeted delivery-related databases, and disease-specific databases.

**TABLE 1:** List of peptide sequences and databases containing their structures.

Database	Peptide type		URL	References
Antimicrobial peptides (AMPs) database				
Peptaibol	Peptaibol	Sequence and structure resource for the unusual class of peptides known as peptaibols	http://www.cryst.bbk.ac.uk/peptaibol	Whitmore and Wallace, 2004
Defensins Knowledgebase	Defensins	A manually curated database of more than 350 defensin records each containing sequence, structure and activity information	http://defensins.bii.a-star.edu.sg	Seebah et al., 2007
PhytAMP	Plant AMPs	A specialized database for plant AMPs containing sequence information and physicochemical or biological data, along with a set of tools for sequence analysis	http://phytamp.hammamilab.org/	Hammami et al., 2009
BACTIBASE	Bacteriocins	A manually curated database of bacterial antimicrobial peptides, along with various tools for bacteriocin analysis, such as homology search, multiple sequence alignments, Hidden Markov Models, molecular modeling	http://bactibase.hammamilab.org/main.php	Hammami et al., 2010
BaAMPs	AMP	A manually curated database of AMPs specifically assayed against microbial biofilms	http://www.baamps.it	Di Luca et al., 2015
APD3	AMP	A AMPs database including a total of 2619 peptides, which currently focuses on natural AMPs with defined sequences and activities and also provides other searchable annotations, including target pathogens, molecule-binding partners, post-translational modifications and animal models.	https://wangapd3.com/main.php	Wang et al., 2016
CAMPR3	AMP	A database of sequences, structures and family-specific signatures of prokaryotic and eukaryotic AMPs containing 10247 sequences, 757 structures and 114 family-specific signatures	www.camp3.bi cnirrh.res.in.	Waghu et al., 2016
InverPep	Invertebrates AMPs	A manually curated database specialized in experimentally validated AMPs from invertebrates and its information	ciencias.medellin. unal.edu.co/gruposde investigacion/prospeccionydisenobiomoleculas/InverPep/public/home_en	Gómez et al., 2017
MBPDB	Milk bioactive peptide	A comprehensive database of bioactive peptides derived from milk proteins from any mammalian source	http://mbpdb.nws.oregonstate.edu	Nielsen et al., 2017
BAGEL4	Bacteriocins	A mining web server of RiPPs (ribosomally synthesized and posttranslationally modified peptides) and bacteriocins	http://bagel4.molgenrug.nl	van Heel et al., 2018
DBAASP v3	AMP	A continuously updated database of antimicrobial activity and structure of peptides	http://dbaasp.org.	Pirtskhalava et al., 2021
Targeted delivery-related databases				
TumorHope	Therapeutic Peptides	A database of experimentally validated tumor homing peptides containing 744 peptides with information on sequence, target tumor, target cell, peptide receptor, techniques of identification, and also providing secondary/tertiary structure, amino acid composition, and physicochemical properties of peptides which derived from their sequences	http://crdd.osdd.net/raghava/tumorhope/.	Kapoor et al., 2012
CPPsite 2.0	Cell penetrating peptide	A manually curated database of cell-penetrating peptides containing around 1850 peptide entries and providing predicted tertiary structure of peptides, possessing both modified and natural residues	http://crdd.osdd.net/raghava/cppsite/	Agrawal et al., 2016
Disease-specific databases				
Hemolytik	Hemolytic and non-hemolytic peptides	A manually curated resource of experimentally determined hemolytic and non-hemolytic peptides containing 3000 entries that include ∼2000 unique peptides with information on sequence, name, origin, reported function, property such as chirality, types (linear and cyclic), end modifications as well as providing predicted tertiary structure of each peptide	http://crdd.osdd.net/raghava/hemolytik/	Gautam et al., 2014
AVPdb	Antiviral peptides	A resource of experimentally verified antiviral peptides targeting over 60 medically important viruses including Influenza, HCV, HSV, RSV, HBV, DENV, SARS, etc. containing detailed information of 2683 peptides, including 624 modified peptides experimentally tested for antiviral activity	http://crdd.osdd.net/servers/avpdb/	Qureshi et al., 2014
ParaPep	Antiparasitic peptides	A repository of experimentally validated antiparasitic peptide sequences and their structures	http://webs.iiitd.edu.in/raghava/parapep/	Mehta et al., 2014
CancerPPD	Anticancer peptides	A manually curated resource of experimentally verified anticancer peptides (ACPs) and anticancer proteins, consists of 3491 ACP and 121 anticancer protein entries, and contains peptides having non-natural, chemically modified residues and D-amino acids	http://crdd.osdd.net/raghava/cancerppd/	Tyagi et al., 2015
SATPdb	Bioactive peptide	A database of structurally annotated therapeutic peptides, holds 19192 unique experimentally validated therapeutic peptide sequences having length between 2 and 50 amino acids, and covers peptides having natural, non-natural, and modified residues	http://crdd.osdd.net/raghava/satpdb/	Singh et al., 2016
THPdb	FDA- approved therapeutic peptides	A manually curated resource of FDA-approved therapeutic peptides and proteins with information on their sequences, chemical properties, composition, disease area, mode of activity, physical appearance, category or pharmacological class, pharmacodynamics, route of administration, toxicity, and target of activity	http://crdd.osdd.net/raghava/thpdb/	Usmani et al., 2017
StraPep	Bioactive peptide	Collection of all the bioactive peptides with known structure, containing 3791 bioactive peptide structures, which belong to 1312 unique bioactive peptide sequences	http://isyslab.info/StraPep	Wang et al., 2018
BIOPEP-UWM	Bioactive peptide	A continuously updated database of bioactive peptides derived from foods	http://www.uwm.edu.pl/biochemia	Minkiewicz et al., 2019
WALTZ-DB 2.0	Amyloid-forming peptide	A database providing information on experimentally determined amyloid-forming hexapeptide sequences	http://waltzdb.switchlab.org/	Louros et al., 2020
Special peptides databases				
NeuroPedia	Neuropeptides	A Neuropeptide Database, provided through hyperlinks to bioinformatic databases on genome and transcripts, protein structure and brain expression of Neuropeptides	www.neuropeptides.nl	Burbach, 2010
NeuroPedia	Neuropeptides	A neuropeptide databank of peptide sequences (including genomic and taxonomic information) and spectral libraries of identified MS/MS spectra of homolog neuropeptides from multiple species	http://proteomics.ucsd.edu/Software/NeuroPedia/	Kim et al., 2011
ConoServer	(Venom toxin peptide) conopeptides	A specialized database of sequence and structures of conopeptides (expressed in carnivorous marine cone snails)	http://www.conoserver.org	Kaas et al., 2012
DADP	Anuran defense peptides	A manually curated resource of anuran defense peptides containing 2571 entries with a total of 1923 non-identical bioactive sequences	http://split4.pmfst.hr/dadp/	Novković et al., 2012
Quorumpeps?	Quorum sensing peptides	A database of quorum sensing peptides with information on structure, activity, physicochemical properties, and related literature	http://quorumpeps.ugent.be/	Wynendaele et al., 2013
NeuroPep	Neuropeptides	A comprehensive and most complete resource of neuropeptides, which holds 5949 non-redundant neuropeptides together with information on source organisms, tissue specificity, families, names, post-translational modifications, 3D structures (if available) and literature references	http://isyslab.info/NeuroPep	Wang et al., 2015
ArachnoServer 3.0	(Venom toxin peptide) (Spider venom)	A manually collection of information on the sequence, structure, function and pharmacology of spider-venom toxin peptides	http://arachnoserver.org/	Pineda et al., 2018
Norin	Non-ribosomal peptides	A database of non-ribosomal peptides together with tools for their analysis containing 1740 peptides	https://bioinfo.cristal.univ-lille.fr/norine/	Flissi et al., 2020

Antimicrobial peptides have garnered a lot of attention for several decades due to their biological activity and their ability to make the pathogens resistant to existing drugs. The AMP databases are the most available among the peptide databases. Some AMP databases are specialized to one type of AMP family, like the Defensins Knowledgebase (defensins) (Seebah et al., 2007), BAGEL4 (bacteriocins) (van Heel et al., 2018), BACTIBASE (bacteriocins) (Hammami et al., 2010), PhytAMP (plant AMPs) (Hammami et al., 2009), the Peptaibol Database (peptaibols) (Whitmore and Wallace, 2004), and InverPep (invertebrates AMPs) (Gómez et al., 2017). Meanwhile, others are general AMP databases, such as APD (Wang et al., 2016), CAMP (Waghu et al., 2016), and DBAASP (Pirtskhalava et al., 2016). APD (antimicrobial peptide database) (Wang and Wang, 2004) initially went online in 2003 with 525 AMPs. It has been extensively accepted and referred to since then. In 2009, an updated version of APD was released (Wang et al., 2009), called APD2, which contains about 1,228 entries and has been consistently updated and further expanded into the APD3 version (Wang et al., 2016). This database is currently focused on collecting natural AMPs with defined sequences and activities. It contains 2,619 AMPs with 261 bacteriocins from bacteria, and 7, 13, 4, and 321 from protists, fungi, archaea, and plants, respectively, with an additional 1,972 animal host defense peptides. Another resource for AMPs is CAMP (collection of antimicrobial peptides) (Thomas et al., 2010). It contains information on sequences of natural as well as synthetic AMPs. The inclusion of the structure of the AMPs and family information constituted the second version of the CAMP (Waghu et al., 2014) known as the CAMPR2. Moreover, CAMPR2 contains the newly identified AMPs sequences. CAMPR3 (Waghu et al., 2016) was introduced in 2016 to include AMP family specific signatures. CAMPR3 provides comprehensive information about the sequences, structures, family signatures, activity profile, sources, target organisms, and hemolytic activity for AMPs, and also links to several external databases. DBAASP (Database of Antimicrobial Activity and Structure of Peptides) was started in 2014 with a collection of published information about the AMPs and the corresponding resources (Gogoladze et al., 2014). In 2016, it was updated to DBAASP v2, with about 8,000 entries, including structural information (Pirtskhalava et al., 2016). It also contributed to the development of several databases such as dbAMP (Jhong et al., 2019), LAMP2 (Ye et al., 2020), PlantPepDB (Das et al., 2020), ADAPTABLE (Ramos-Martín et al., 2019), and starPepDB (Aguilera-Mendoza et al., 2019). Most recently, another updated version of DBAASP (DBAASP v3) (Pirtskhalava et al., 2021) has been released to include new content and additional user services. This database has continuously developed its predictive tools to be employed in the *de novo* design of peptide-based drugs. Its efficacy for the design of peptide-based antimicrobial agents against both gram-positive and gram-negative bacteria has been experimentally confirmed (Vishnepolsky et al., 2019a, b). The BaAMPs database was developed for AMPs with the property of disrupting microbial biofilms (Di Luca et al., 2015). MBPDB is a database of the bioactive peptides of milk origin (Nielsen et al., 2017). CPPsite and TumorHoPe are targeted delivery-related databases. CPPsite (Gautam et al., 2012) is the first database of CPPs that contains 843 entries along with the sequence information, subcellular localization, physicochemical properties, and uptake efficiency. The updated version of CPPsite (Agrawal et al., 2016), called CPPsite 2.0, contains 1,850 entries, including the model system, cargo information, chemical modifications, predicted tertiary structure, and other information. The TumorHoPe database (Kapoor et al., 2012) contains 744 peptides that can recognize tumor tissues and tumor-associated microenvironments.

Disease-specific databases encompass peptides that can be used to design therapeutic peptides capable of targeting specific diseases. PDB (Usmani et al., 2017) is a database of FDA-approved peptides and protein therapeutics, CancerPPD (Tyagi et al., 2015) is a database of anticancer peptides (ACPs) and proteins, and AntiTbPdb (Usmani et al., 2018) is a database of experimentally verified anti-mycobacterial and anti-tubercular peptides. The HIPdb database was established to provide information about 981 HIV-inhibiting peptides (Qureshi et al., 2013), including their sequences and half-maximal inhibitory concentrations (IC_50_). AVPdb contains 2,683 antiviral peptides and the data about their sequences, efficacy, modifications, and predicted structures (Qureshi et al., 2014). ParaPep (Mehta et al., 2014) is a database of experimentally validated anti-parasitic peptide sequences and their structures curated and compiled from literature, patents, and various other databases. This database was created by Mehta et al. (2014) and contains 863 entries that include 519 unique peptides whose anti-parasitic activities were evaluated against multiple species of *Plasmodium*, *Leishmania*, and *Trypanosoma*. In ParaPep, the structures of peptides consisting of natural, and modified, amino acids have been predicted using the PEPstr software. The Hemolytik database (Gautam et al., 2014) is an information system for experimentally determined hemolytic and non-hemolytic peptides obtained by manual extraction from numerous scientific papers and various databases. This database was created by Gautam et al. (2014) and contains 3,000 entries that include 2,000 unique peptides whose hemolytic activities were assessed on erythrocytes isolated from as many as 17 different sources. WALTZ-DB (Beerten et al., 2015) is the largest available database of experimentally characterized amyloid-forming short sequences. This open-access database was created by Beerten et al. (2015). It contains 1,089 entries that provide primary information about amyloid aggregation incorporated in related databases. Louros et al. (2020) released an updated and significantly expanded version of this database, called WALTZ-DB 2.0. With WALTZ-DB 2.0, the structural model and information were added to the entries. The 3D models of the amyloid fibril cores of the entries were generated using a computational methodology developed in the Switch lab. It also provides a user-friendly option for data filtering and browsing. BIOPEP (Minkiewicz et al., 2008; Iwaniak et al., 2016), currently BIOPEP-UWM (Minkiewicz et al., 2019), is a widely used resource for the identification of bioactive peptides and bioactivity prediction as well as for *in silico* approaches.

The structural information of bioactive peptides is essential for the development of peptide-based drugs. Two databases, namely StraPep and SATPdb, are structural databases that are dedicated to the collection of bioactive peptides with known structures. StraPep (Wang et al., 2018) is dedicated to collecting all of the bioactive peptides with known structures. It displays the structures for 3,791 peptides and provides detailed information for each one (i.e., post-translational modification, experimental structure, secondary structure, the location of disulfide bonds, etc.). SATPdb (Singh et al., 2016) is a database of structurally annotated therapeutic peptides. It has been curated from 22 public peptide databases and has 19,192 unique, experimentally validated therapeutic peptide sequences. Several databases have been designed for unique peptides as well. Quorumpeps (Wynendaele et al., 2012) is developed for quorum sensing peptides, ConoServer (Kaas et al., 2012) and ArachnoServer (Pineda et al., 2018) are focused on venom toxin peptides; NORINE (Flissi et al., 2020) contains information on non-ribosomal peptides; DADP (Novković et al., 2012) is a database of defense peptides; and the NeuroPep, Neuropedia, and Neuropeptides^1^ databases contain information on neuropeptides. Besides, databases covering proteins are also considered valuable resources for peptides; for example, UniProt (Universal Protein Resource)^2^, which is a central resource of protein data, and protein structure databases such as PDB^3^ (Abes et al., 2007), which contain the 3D structure of biomolecules.

## *In Silico* Tools and Algorithms for Peptide Design

Peptides are the most amenable modulators that can be used to tackle the high surface area of PPIs. They can easily be synthesized, closely mimic the principal features of a protein, and be modified to attain higher stability, bioavailability, and binding strength. Sequence-based design and structure-based design are two major approaches for peptide design. Sequence-based peptide designing involves various physiochemical properties of the peptides and optimizing the peptide stability, toxicity, immunogenicity, and antibody specificity. In this regard, a plethora of *in silico* tools have been developed for sequence-based designing of novel peptides with therapeutic properties ranging from cell-penetrating to anti-microbial, anti-parasitic, anti-cancer, and anti-hypertension (Table 2).

**TABLE 2:** Sequence-based peptide design tools.

Description	Name URL	Method	References
Prediction of AMPs	APD3: https://wangapd3.com/main.php	Support vector machine	Wang et al., 2016
	CAMPR3: http://www.camp3.bicnirrh.res.in/prediction.php	[Support Vector Machines (SVMs), Random Forests (RF) and Discriminant analysis (DA)]	Waghu et al., 2016
	Deep-AmPEP3: https://cbbio.cis.um.edu.mo/AxPEP	Optimal feature set of PseKRAAC reduced amino acids composition and convolutional neural network	Yan et al., 2020
Prediction of Antiviral peptide	AVPpred: http://crdd.osdd.net/servers/avppred	Support Vector Machine	Thakur et al., 2012
	AVCpred: http://crdd.osdd.net/servers/avcpred	Support vector machine	Qureshi et al., 2017
	Meta-iAV http://codes.bio/meta-iavp/	Sequence-based meta-predictor	Schaduangrat et al., 2019b
Prediction of Antifungal Peptides	Antifp: http://webs.iiitd.edu.in/raghava/antifp	Support vector machine based model developed using compositional features of peptides	Agrawal et al., 2018
Prediction of antibacterial peptide	Antibp2: http://www.imtech.res.in/raghava/antibp2/	Support Vector Machine (SVM)	Lata et al., 2010
Prediction of Anti-Tubercular Peptides	AtbPpred: http://thegleelab.org/AtbPpred	Two-layer machine learning (ML)-based predictor	Manavalan et al., 2019a
Prediction and classification of antimicrobial peptides	ClassAM: http://www.bicnirrh.res.in/classamp/	Support vector machine	Joseph et al., 2012
Prediction of antimicrobial peptides	iAMPpred: http://cabgrid.res.in:8080/amppred/	Support vector machine	Meher et al., 2017
Anticancer peptide prediction	iACP: https://bio.tools/iacp	Support vector machine (SVM)	Chen et al., 2016
	MLACP: http://www.thegleelab.org/MLCPP/	Support vector machine- and random forest-based machine-learning methods	Manavalan et al., 2017
	ACPred: http://codes.bio/acpred/	Machine learning models (support vector machine and random forest) and various classes of peptide features	Schaduangrat et al., 2019a
	AntiCP 2.0: https://webs.iiitd.edu.in/raghava/anticp2	Various input features and implementing different machine learning classifiers	Agrawal et al., 2020
Prediction and Analysis of Anti-Angiogenic Peptides	TargetAntiAngio: http://codes.bio/targetantiangio/	Random forest classifier in conjunction with various classes of peptide features.	Laengsri et al., 2019
Prediction of anti-hypertensive peptides	mAHTPred: http://thegleelab.org/mAHTPred	Six different ML algorithms, namely, Adaboost, extremely randomized tree (ERT), gradient boosting (GB), k-nearest neighbor, random forest (RF), and support vector machine (SVM) using 51 feature descriptors derived from eight different feature encoding	Manavalan et al., 2019b
Half Life Prediction	HLP: http://www.imtech.res.in/raghava/hlp/	SVM based models	Sharma et al., 2014
Predict and design toxic/non-toxic peptides	ToxinPred: https://webs.iiitd.edu.in/raghava/toxinpred	Machine learning technique and quantitative matrix using various properties of peptides	Gupta et al., 2013
Improved and robust prediction of hemolytic peptide and its activity	HLPpred-Fuse: http://thegleelab.org/HLPpred-Fuse/	Integrating six different machine learning classifiers and nine different sequence-based encoding	Hasan et al., 2020
Prediction of pro-inflammatory antigenicity of peptides	ProInflam: http://metagenomics.iiserb.ac.in/proinflam/	Machine learning-based prediction	Gupta et al., 2016
Prediction of Cell penetrating peptide	CellPPD : http://webs.iiitd.edu.in/raghava/cellppd/	Support vector machine and motif 2013 based	Gautam et al., 2013
	CPPpred: http://bioware.ucd.ie/cpppred	Neural networks	Holton et al., 2013
	CPPred-RF: http://server.malab.cn/CPPred-RF	Two-layer prediction framework based on the random forest algorithm	Wei et al., 2017
Prediction of bioactive peptide	PeptideRanker: http://bioware.ucd.ie/	Neural Network	Mooney et al., 2012

Numerous methods, which are classified into general and specific methods, have been developed to predict AMPs. The APD3 (Wang et al., 2016) and CAMPR3 (Waghu et al., 2016) databases are among the general method predictors. They are designed to predict whether a given peptide is AMP or non-AMP. APD3 is developed for the classification, prediction, and design of AMPs using the parameter space defined by all available natural peptides in the database. The CAMPR3 implements four different machine-learning (ML) techniques to develop a peptide model. Deep-AmPEP30 (Yan et al., 2020) is a recently developed method that is used to predict bioactive sequences from genomes. This tool uses a short-length AMP prediction method based on the optimal feature set of PseKRAAC, reduced amino acid composition, and convolutional neural networks. The second group of methods is designed to predict AMPs, specifically on viruses, fungi, bacteria, or parasites. AVPpred (Thakur et al., 2012), AVCpred (Qureshi et al., 2017), and Meta-iAV (Schaduangrat et al., 2019b) are the more commonly used tools for the prediction of antiviral peptides. AVPpred (Thakur et al., 2012) is a web server used for the collection and detection of highly effective antiviral peptides (AVPs) using ML techniques such as support vector machine (SVM), features like the amino acid composition, and physicochemical properties. The AVCpred method is an SVM-based AVP prediction method. The experimental inhibitory percentage from ChEMBL (a large-scale bioactivity database for drug discovery) predicts the antiviral compounds against HIV, hepatitis C virus, hepatitis B virus, human herpesvirus, and 26 other viruses. Meta-iAVP is a sequence-based meta-predictor with an efficient feature representation (Schaduangrat et al., 2019b). It is designed for the accurate prediction of AVPs from peptide sequences. Antifp (Agrawal et al., 2018) is designed to predict antifungal peptides using features like amino acid composition, and similarly, Antibp2 (Lata et al., 2010) is another SVM-based method developed to predict antibacterial peptides. AtbPpred (Manavalan et al., 2019a) is a two-layer ML-based predictor for the identification of anti-*Mycobacterium tuberculosis* peptides. Moreover, ClassAMP (Joseph et al., 2012) and iAMPpred (Meher et al., 2017) are two methods used to predict the AMP class (e.g., antibacterial, antifungal, and antiviral). iAMPpred (Meher et al., 2017) predicts the probability of a peptide to be an antibacterial, antifungal, and antiviral agent by providing the probability score for all of the three classes.

The discovery of ACPs has provided an alternative approach to treating cancer. iACP (Chen et al., 2016), MLACP (Manavalan et al., 2017), ACPred (Schaduangrat et al., 2019a), and AntiCP 2.0 (Agrawal et al., 2020) are some famous *in silico* tools for the prediction and design of ACPs. The discovery of anti-angiogenic peptides is a promising therapeutic route for cancer treatment. TargetAntiAngio (Laengsri et al., 2019) was developed for the prediction and characterization of anti-angiogenic peptides using the random forest classifier in conjunction with various classes of peptide features and was demonstrated to be superior to other existing methods. mAHTPred (Manavalan et al., 2019b) was developed to predict anti-hypertension peptides using six different ML algorithms and showed superior performance compared to existing methods. HLP (half-life prediction) (Sharma et al., 2014) was developed for the prediction and design of peptides with desired half-life using SVM methods.

ToxinPred (Gupta et al., 2013) is one of the most applied SVM-based tools for predicting peptides toxicity. HLPpred-Fuse (Hasan et al., 2020) is the only tool that simultaneously identifies hemolytic peptides and their activities. It has fused six different ML classifiers in a robust hemolytic peptide prediction method. ProInflam (Gupta et al., 2016) was developed to predict the pro-inflammatory antigenicity of peptides using an ML-based prediction tool. CellPPD (Gautam et al., 2013) is an SVM-based method that has been widely used to predict CPPs. CPPpred (Holton et al., 2013) is another server used to predict CPPs based on artificial neural networks. In CPPred-RF (Wei et al., 2017), the random forest algorithm can simultaneously predict the CPPs and their uptake efficiency. PeptideRanker (Mooney et al., 2012) is also a webserver used to predict the probability of the peptides being bioactive and ranks the bioactive peptides.

## *In Silico* Tools and Algorithms for Peptide Modeling

Bioactive peptides are critical in industrial, medical, and biological applications, and they play pivotal roles in regulating various biological processes (Gesell et al., 1997; Liu et al., 2008). There are several conventional methods to identify the tertiary structure of peptide molecules, including CD, electron paramagnetic resonance (EPR), Fourier-transform infrared (FTIR), and NMR spectroscopies and x-ray crystallography. However, these empirical methods are labor-intensive, time-consuming, and expensive to perform. Moreover, the employed solvent may have a significant influence on the peptide structure in some instances. Given these circumstances, computational methods for predicting the 3D structures of the peptide have emerged to circumvent these limitations. These methods are expected to contribute significantly to the delineation of peptide sequence to function relationships and the promotion of efficient designs for new peptide molecules. Like the methods for predicting protein 3D structure, peptides could be modeled by employing the homology, threading, and *ab initio* approach. The peptide modeling tools use one or a combination of these methods to predict the 3D structures of the peptides. The homology and threading methods rely on suitable template structures. Previously resolved peptide structures stored in PDB have a crucial influence on the accuracy of homology-based peptide modeling (Sali, 1995; Sanchez and Šali, 1997). The threading method is performed using the existing folds of proteins as the modeling templates (Bowie et al., 1991; Jones et al., 1992). Unlike the homology and threading methods, the *ab initio* approach is not template-based and exploits the physicochemical features to predict low-energy folding of the peptides (Monge et al., 1994; Bradley et al., 2005). In this regard, the bioinformatic tools involved in peptide modeling are classified into template-based and template-free methods. Various tools and servers have been developed for the prediction of peptide structures. Among the various available tools, six highly referred servers (PEPstr, Protinfo, PEP-FOLD, PEP-FOLD3, Hmmstr/Rosetta, PepLook, and PepSite & FlexPepDock server) are introduced in the following sections.

### PEPstr Server

This server can predict the 3D structures of the peptide with an average length of 7–25 and sometimes 27 amino acids. It uses valuable and vital information about the beta-turns in predicting the 3D structure of the peptide. This server acts through different steps to model the peptide 3D structure. Primarily, all residues of the peptide get an extended conformation (phi = Psi = 180°). The secondary structure information from regular secondary structures (helices, beta-strands, and coil) and the beta-turns method help to detect the 2D structure of the peptide. Then, the conformational shape is created by assigning the Psi (Ψ) and phi (Φ) angles of the main chain. The standard Dunbrack backbone-dependent Rotamer library is used to determine the side chain angles. Ultimately, the obtained peptide model is refined by molecular dynamics (MD) simulation and energy minimization. The modeled 3D structure of the peptide will be stored in PDB format. Of note is that MD simulation can be accomplished in vacuum, hydrophilic, and hydrophobic states. The web server of PEPstr^4^ was evaluated for the modeling of short peptides (Kaur et al., 2007). This server can model natural and non-natural amino acids, D amino acids, terminal modifications, peptide cyclization, post-translational and advanced modifications of residues, and structure simulations.

### Protinfo Server

This server is highly suitable for the prediction of complicated protein structures. The function of this server is based on the interolog approach for the detection of experimental samples. Using the interolog approach is helpful for the identification of similar results found in the existing databases. Following this step, this server modeled input queries based on the determined homologous samples. One of the prominent features of the Protinfo server is that it can support a wide range of templates, from small amino acid subunits to a large number of them. The Protinfo PPC web server is available at http://protinfo.compbio.washington.edu/ppc/ (Hung et al., 2005).

### PEP-FOLD Server

This is a high-performance server for *de novo* prediction of peptide structures from amino acid sequences. It utilizes a hidden Markov model (HMM) to make the predictions. It functions through the identification of primary structural alphabet (SA) letters in each sequence. The SA letters extracted by the HMM are necessary to describe the correct conformations of four consecutive residues (Zemla, 2003). It couples the predicted series of SA letters to a greedy algorithm and a coarse-grained force field (Souza et al., 2020). PEP-FOLD can handle peptides with 9–25 amino acids (Maupetit et al., 2009, 2010). PEP-FOLD3 is an improved version of the PEP-FOLD server. It is also a *de novo* approach with a more advanced and faster peptide structure modeling system. It can accommodate a vast range of peptide sizes ranging from 5 to 50 amino acids (Lamiable et al., 2016). PEP-FOLD and PEP-FOLD3 are respectively accessible at https://bioserv.rpbs.univ-paris-diderot.fr/services/PEP-FOLD/ and https://bioserv.rpbs.univ-paris-diderot.fr/services/PEP-FOLD3/.

### I-Sites/Hmmstr/Rosetta Server

This server can predict the secondary, local, super-secondary, and tertiary structures of the protein sequences. This server also uses a hidden Markov model (HMMSTR) for local and secondary structure prediction, based on the I-sites library. It has three parts: I-site, Hmmstr, and Rosetta. I-site-motifs is a library that contains an extensive collection of small motif sequences (3–19 motifs). The I-site library is connected with numerous structured databases and encompasses about one-third of database sequences (Bystroff et al., 2000). According to the literature, the combination of I-sites and Rosetta is advantageous. Rosetta *ab initio* 3D predictor is a Monte Carlo (MC) Fragment Insertion protein-folding program that can use the results of Critical Assessment of Fully Automated Structure Prediction (CAFASP2) during the process (Fischer et al., 2001). There are some steps in the execution process of this server. The first step is the creation of a profile sequence through multiple alignments of the input sequence using PSI-BLAST (Position-Specific Iterative Basic Local Alignment Search Tool). The second step is the prediction of I-site motifs with a determined profile sequence. The third step is the preparation of fragment movesets from predicted I-site motifs. The fourth step is the modeling of secondary and local structures *via* HMMstr or the analysis of fragment moveset by the Rosetta server (Bystroff and Shao, 2002). Moreover, the I-sites/Hmmstr/Rosetta web server^5^ is developed for 3D prediction.

### PepLook Server

PepLook is another 3D peptide modeling web server that can model sequences with more than 30 residues. This server prepares many randomly produced peptide structures by altering the SA angles (Φ/Ψ). Modeling cyclic peptide conformation using distance restraint is one of the vital beneficial aspects of this server (Etchebest et al., 2005). Moreover, this server can model the 3D structure of post-translationally modified amino acids (carboxylated or hydroxylated), synthetic amino acids, and ribosomal peptides. PepLook can measure the energy factor of some features, including internal and external hydrophobicity, complete peptide structures (all atoms), and electrostatic and van der Waals interactions (Thomas et al., 2006). The PepLook server could be found at https://orbi.uliege.be/handle/2268/135016.

### PepSite and FlexPepDock Server

Protein–peptide interactions are a vital part of many cellular signaling pathways. Getting a good grasp of these interactions would bring about a mechanistic understanding of how cell networks are regulated. The PepSite server can predict the binding of a given peptide onto a protein structure which unveils the details of the interaction of interest. PepSite 2^6^ is a complete rewrite of the original PepSite, which can speed up the presentation of results to a fraction of a second. The surface position-specific scoring matrix (S-PSSMs) algorithm is used in this server to detect the binding sites for each peptide residue. Ultimately, a suitable peptide sequence can be generated against predicted biding sites considering certain distance limitations (Trabuco et al., 2012). FlexPepDock^7^ is a high-resolution peptide-protein docking (refinement) protocol for the modeling of peptide–protein complexes. It is implemented in the Rosetta framework, which can be combined with the result of PepSite to design atomic models for given peptides in the vicinity of binding sites (London et al., 2011; Raveh et al., 2011). The summary of the information on the mentioned servers is presented in Table 3.

**TABLE 3:** List of important *in silico* peptide modeling servers.

Type	Brief description	URL	References
Hmmstr/Rosetta	The HMMSTR/Rosetta Server predicts the structure of proteins from the sequence: secondary, local, supersecondary, and tertiary	http://www.bioinfo.rpi.edu/~bystrc/hmmstr/about.html	Bystroff et al., 2000; Bystroff and Shao, 2002
Protinfo	Protinfo PPC is a web server that predicts atomic level structures of interacting proteins from their amino-acid sequences	http://protinfo.compbio.washington.edu/ppc/	Hung et al., 2005; Kittichotirat et al., 2009
Pepstr	This server predicts the tertiary structure of short peptides with sequence length varying between 7 to 25 residues	http://www.imtech.res.in/raghava/pepstr/	Kaur et al., 2007
FlexPepDock	The Rosetta FlexPepDock protocol for high-resolution docking of flexible peptides which mainly consists of two alternating modules that optimize the peptide backbone and rigid body orientation, respectively, using the Monte-Carlo with Minimization approach.	http://flexpepdock.furmanlab.cs.huji.ac.il/	Raveh et al., 2010
PepLook	Peplook server, an efficient tool to predict peptide conformation	https://orbi.uliege.be/handle/2268/135016	Beaufays et al., 2012
PepSite	This server is a tool for accurate prediction of peptide binding sites on protein surfaces	http://pepsite2.russelllab.org	Trabuco et al., 2012
Pep-Fold	PEP-FOLD is a *de novo* approach aimed at predicting peptide structures from amino acid sequences. This method, based on structural alphabet letters	https://bioserv.rpbs.univ-paris-diderot.fr/services/PEP-FOLD/	Maupetit et al., 2009; Thevenet et al., 2012
Pep-Fold3	PEP-FOLD3 latest evolution comes with a *new 3D generation engine*, based on a new Hidden Markov Model sub-optimal conformation sampling approach, faster by one order of magnitude than the previous greedy strategy, while not affecting performance	https://bioserv.rpbs.univ-paris-diderot.fr/services/PEP-FOLD3/	Lamiable et al., 2016

## *In Silico* Tools for the Prediction of Protein–Protein Interfaces

**FIGURE 1:**
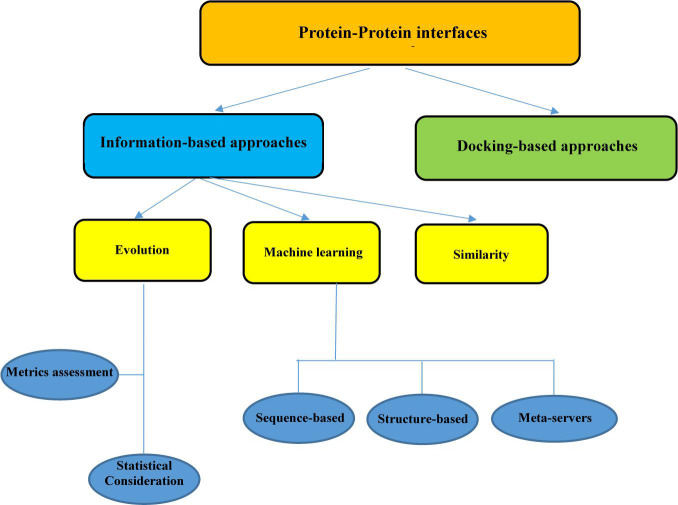
Summary of protein–protein interface analysis procedure.

Proteins are vital agents that carry out all biological activities in the cells. PPIs play a pivotal role in the execution of these functions. Hence, investigating the changes that occur in the space between two proteins can be highly beneficial in the elucidation of disease etiology and the adaptation of appropriate treatment strategies. Various experimental methods exist for detecting biological changes in the PPI interface, including NMR, x-ray crystallography, mass spectrometry, and alanine scanning mutagenesis. NMR and x-ray crystallography can identify the interfaces at the atomic level, while the other methods like alanine scanning act at the residue level. Although the information about some of the PPI interface networks is extracted from high-throughput experiments, these experimental methods have their constraints mainly because they are expensive and time-consuming (Chen and Liu, 2005; Shoemaker and Panchenko, 2007; Casals et al., 2012; Koboldt et al., 2013). In this study, we focus on some computational methods for predicting the properties of the PPIs that can be valuable in circumventing the limitation of traditional pipelines. There are various computational approaches for the analysis of PPI interfaces. The existing strategies could be divided into information-based approaches and docking-based approaches (Figure 1). Docking is an important method that can facilitate the reconstruction of binary residue connections between two protein sections.

### Information-Based Approach

A wide variety of *in silico* methods has been developed for data-based approaches. This approach is instituted on the information that is extracted from the results of conventional experiments. Three main strategies have been established for data-based approaches to analyze the PPI interfaces, namely the similarity-based method, the ML method, and the evolutionary-based method.

#### Similarity-Based Method

This method, also known as the “template-based” method, is one of the most widespread searching approaches for annotating the genome function. Instead of using partial sequence and statistical features of the sequence, the method relies on the similarity of whole protein sequences. Several biological properties can be obtained by multiple sequence alignments that are practical for identifying PPIs. These properties include the conservation of amino acids, the similarity between interfacing proteins, and gene fusion. Different behaviors can be expected from the proteins using this method. For example, unstable proteins tend to utilize different interfacial binding patterns. Therefore, using homology search for the extraction of interface residues remains a controversial issue (Grishin and Phillips, 1994; Caffrey et al., 2004; Esmaielbeiki et al., 2016). A list of similarity-based prediction tools for PPI interfaces is represented in Table 4.

**TABLE 4:** Similarity-based prediction databases (information-based approach).

Type	Brief introduction	URL	References
IBIS	IBIS is practical server that can sever and identify most of interaction (protein-protein, RNA-protein and etc.)	http://www.ncbi.nlm.nih.gov/Structure/ibis/ibis.cgi	Shoemaker et al., 2010
PredUS	PredUS is a structural similarity-based method and it is useful for detection of several features including designing of adjacent protein, mapping the interfacial space residues, measurement of residue interface score	https://bhapp.c2b2.columbia.edu/PredUs/	Zhang et al., 2011
PrISEC	This server measures surface patch which its function algorithm based on surface residues and atomic formation.	http://prise.cs.iastate.edu/	Jordan et al., 2012
PS-HomPP*	Extracting of interfacial residues via similar residues between binding proteins(intelligent server)	http://ailab1.ist.psu.edu/PSHOMPPIv1.2/	Esmaielbeiki and Nebel, 2014
NPS-HomPPI*	Prediction of probable interacting residues with different proteins(unintelligent server)	http://ailab1.ist.psu.edu/NPSHOMPPI/	Esmaielbeiki and Nebel, 2014
ProBiS	Using local structure alignment for identification of protein-protein interfaces	http://probis.cmm.ki.si/	Esmaielbeiki et al., 2016

* Intelligent server means a server that is considered protein with special connector. *Unintelligent server means a server that is considered protein with different connectors.

#### ML Method

Although there are beneficial aspects for the similarity-based method of predicting PPIs, it suffers from restrictions such as lack of accessible interfaces for experimental homologs and low quality (de Vries and Bonvin, 2008). ML methods operate based on a comparative pattern between interfacial protein residues and non-interfacial residues. This method could compensate for the limitations of the similarity-based methods. This approach can be categorized within the sequence and structural-based methods and consensus identifiers (Xie et al., 2020). Moreover, the use of ML methods for the prediction of PPIs has represented improved performance in comparison to other conventional approaches, such as neural networks. Thus, ML methods could play a significant role in therapeutic peptide development (Yang, 2008).

##### Sequence- and structural-based method

The sequence-based interface identifiers operate according to the features of the protein sequences. Most of these tools rely on evolutionary information from multiple sequence alignments (MSA) projected on the protein surfaces (Jordan et al., 2012). Therefore, these methods are inadequate in detecting interfaces of proteins with sparse homolog sequences and evolutionary variable regions. Advancements in structural proteomics have justified the establishment of structure-based automated methods for the prediction of functional surfaces of proteins (Jordan et al., 2012). The performance of this method depends on two 3D mappings and the 3D identifier. The 3D mapping is pertinent to the detection of close structures, and the 3D identifier is related to specific features such as protein surface accessibility (Hoofnagle et al., 2003; Heinig and Frishman, 2004), B-Factor (Halabi et al., 2009), the formation of the protein surface, and the 2D structure (Heinig and Frishman, 2004; Hamer et al., 2010) (Table 5).

**TABLE 5:** Sequence- and structural-based databases (ML approach).

Type	Brief introduction	URL	References
ProMate(Str)*	Circling of surface residues and Possible estimation of the binding affinity in each selected residues	bioinfo41.weizmann.ac.il/promate/promate.html	Neuvirth et al., 2004
WHISCY	It is a versatile server that is capable to use sequence and structure and its function is measurement of similarity score based on Dayhoff matrix	http://nmr.chem.uu.nl/Software/whiscy/	de Vries et al., 2006
PINUP(Str)*	Presentation of scoring function such as conservation score	http://sysbio.unl.edu/services/PINUP/	Liang et al., 2006
PIER(Str)*	Recognition of interfacial & un-interfacial surface residues	http://abagyan.ucsd.edu/PIER/	Kufareva et al., 2007
SPPIDER (Str)*	Identification of measured (RSA)* and real (RSA)	http://sppider.cchmc.org/	Porollo and Meller, 2007
PSIVER(Seq)*	Detection of binding sites between two proteins via Naïve Bayes and PSSM	http://tardis.nibio.go.jp/PSIVER/	Murakami and Mizuguchi, 2010

(Str)*, structure; (Seq)*, sequence; (RSA)*, relative solvent accessibility.

##### Meta-servers

Meta-servers combine several interface prediction methods into a consensus predictor to attain more reliable and stable predictions compared to the results of each predictor on its own. This means that meta-servers can act creatively to use different databases as complementary units to improve their function (Kozlowski and Bujnicki, 2012). Three common meta-servers are presented in Table 6.

**TABLE 6:** Three main meta-servers for prediction of interfaces (ML approach).

Type	Brief introduction	URL	References
Cons-PPISP(Str)	A server that is able to identify interaction sites by using some features such as specific position of surface residue, accessibility of surface residue	https://pipe.scs.fsu.edu/ppisp.html	Chen and Zhou, 2005
meta-PPISP(Str)	Strength incorporation of recognizing sections of three servers (PINUP, Cons-PPISP, ProMate)	http://pipe.scs.fsu.edu/meta-ppisp.html	Qin and Zhou, 2007
CPORT(Str)	Incorporation of six servers (PINUP, Cons-PPISP, ProMate, SPPIDER, PIER, WHISCY)	http://alcazar.science.uu.nl/services/CPORT/	de Vries and Bonvin, 2011

#### Evolutionary-Based Approach

The prediction of PPI interfaces using evolutionary data is one of the most well-known interface classifiers. This approach is deemed as a realistic approach for the identification of the biological features of PPI interfaces. The EPPIC (Evolutionary Protein–Protein Interface Classifier) server^8^ is a tool designed to predict the quaternary structure of proteins from their crystal structures. Primarily, it classifies the interfaces of the crystal structure to determine their biological relevance. All topologically valid assemblies are computed, and the individual interface scores are used to predict the most likely quaternary assembly (Duarte et al., 2012).

### Docking-Based Approach

Docking is one of the most well-known computational methods that are highly advantageous for predicting interfaces of biological targets and molecules (Kitchen et al., 2004). This method is practical for atomic-level investigation of molecular interactions. There are several different docking methods, and the selection of each server depends on the complexity of the problem and structure sources. Some structures are extracted *via* NMR or x-ray crystallography, which are suitable for the available docking servers. However, the template-based approach is the best candidate for predicting complex structures (Porter et al., 2019). Testing the accuracy of computational docking algorithms in blind predictions is managed by the CAPRI (Critical Assessment of Predicted Interactions) project (Janin et al., 2003). Molecular docking can be accomplished by a vast range of tools that are already accessible. Many docking tools act on the principle of rigid-body interactions, which results in a suitable match between the surfaces of tertiary (3D) structures. However, working with them has many restrictions such as protein flexibility, which is not considered. Given the importance of molecular docking in the determination of interface residues, many efforts have been made to mend the existing limitations (Luiz Folador et al., 2015; Pinzi and Rastelli, 2019).

## *In Silico* Hotspot Prediction Tools

It is well established that the energy distribution is not uniform in PPI interfaces, and a small number of residues have the largest share in binding free energy. Wells and Clackson, who studied the binding of the growth hormone to its receptor, discovered these residues and used the term hotspot for them (Wells, 1991; Clackson and Wells, 1995; Schreiber and Fersht, 1995; Stites, 1997; Bogan and Thorn, 1998; Clackson et al., 1998; Hu et al., 2000; Keskin et al., 2005; Kouadio et al., 2005). Subsequent studies showed that hotspots form a small number (about 9.5%) of residues of the interface area. Therefore, a more precise definition of hotspots has been proposed, which states that a hotspot is a residue whose mutation to alanine reduces at least 2 kcal/mol in binding free energy (ΔΔG = ΔG ^*mutant type*^ –ΔG^ wild type^) (Bogan and Thorn, 1998; Thorn and Bogan, 2001; Moreira et al., 2007). Hotspot residues are mainly composed of tyrosine (12.3%), arginine (13.3%), and tryptophan (21%) amino acids (Lichtarge et al., 1996; Bogan and Thorn, 1998). Studies have shown that hotspots have conserved structures and predictable physicochemical properties (DeLano, 2002; Moreira et al., 2007; Kenneth Morrow and Zhang, 2012). Impaired PPIs can cause many diseases, such as neurological disorders and cancer. Moreover, the conserved structure of hotspots, as well as their great impact on the binding energy, has made them attractive medical targets for the design of inhibitor drugs. Thus, many unwanted PPIs can be avoided by the use of these inhibitors, and they could be more effective in treating various diseases (Livnah et al., 1996; Wrighton et al., 1996; Tilley et al., 1997; Li et al., 1998; DeLano et al., 2000; Sidhu et al., 2000; Arkin and Wells, 2004; Thanos et al., 2006; Moreira et al., 2007; Wells and McClendon, 2007; White et al., 2008; Blazer and Neubig, 2009). As mentioned, hotspots are predictable, and it is worth mentioning that one of the prediction methods is based on the experimental alanine scanning method. For example, when a large tryptophan residue (as one of the three main hotspot residues) mutates into alanine, this difference in size causes the formation of a cavity, resulting in instability of the complex due to binding energy reduction. Alanine scanning means that when a residue mutates into alanine, the amount of binding energy decreases; if this decreased energy is significant (10-fold or more), then that mutated residue is considered as a hotspot (Bogan and Thorn, 1998; DeLano, 2002). Mutation to alanine residue removes the side chain effect. The methyl side chain of the alanine residue is relatively neutral and also lacks the additional flexibility contribution (Cunningham and Wells, 1989; Wells, 1990; Skolnick et al., 2000). Although glycine mutagenesis removes the contribution of the side chain, it can cause flexibility in the protein backbone (Morrison and Weiss, 2001). Hence, glycine mutagenesis is not considered for hotspot detection. The Alanine Scanning Energetics Database (ASEdb) contains the results of alanine scanning experiments. The Binding Interface Database (BID) has verified experimental hotspots in the literature (Thorn and Bogan, 2001; Fischer et al., 2003). Despite their advantages, these databases also are associated with several drawbacks. The hotspots obtained from experimental studies can only be attributed to a limited number of complexes. Moreover, it is recommended to avoid these data to interpret specific residual interactions (DeLano, 2002). Experimental mutagenesis of proteins to find hotspots is not practical and useful on a large scale because individual mutated proteins must be purified and analyzed separately. It should also be noted that alanine scanning and other experimental hotspot analysis methods are highly time-consuming and costly. Given these circumstances, theoretical and computational prediction methods seem attractive for hotspot residue prediction (Kenneth Morrow and Zhang, 2012). FoldX^9^ is a hotspot prediction tool and server which uses the FOLDEF algorithm developed by Guerois et al. (2002). This server predicts the hotspots of PPIs by using an energy-based method. It finds the PPI energy changes with the computational alanine scanning technique (Schymkowitz et al., 2005; Kenneth Morrow and Zhang, 2012). The Robetta server^10^ developed by Baker and Kortemme includes various parameters such as implicit solvation and hydrogen bonding, packing interactions, solvation interactions, and Lennard-Jones interactions to calculate the interaction free energy. The method employed in this server is similar to that in FoldX (energy-based method), and the technique used is computational alanine scanning (Kim et al., 2004). Similar to FoldX, the parameters obtained from the Robetta server are based on changes in protein stability. This server mutates the side chains to alanine and then locally repacks the parts of the structure that fall in a 5-Å radius of the mutated residue. The rest of the protein structure remains unchanged. The changes in binding energies of PPIs result from these mutations and form the basis for hotspot predictions (Kortemme et al., 2004). The Robetta server can accurately predict 79% of hotspot residues with a 1.0 kcal/mol cutoff. However, it enables us to find the hotspots involved in hydrogen bonds with water molecules (Kenneth Morrow and Zhang, 2012). PP_Site is a structure-based tool with a simple algorithm technique based on three factors, namely van der Waals interactions, hydrophobic interactions, and H-Bond, developed by Gao et al. (2004) and Kenneth Morrow and Zhang (2012). FTMAP^11^ is an energy-based hotspot prediction tool and server. It uses a probe-based rigid-body docking with fast Fourier transform correlation. The users of FTMAP only need the PDB code or the PDB file of the protein for the prediction (Brenke et al., 2009; Kenneth Morrow and Zhang, 2012). PCRPi (Presaging Critical Residues in Protein interfaces) is a method that uses the Bayesian networks technique to unify evolutionary, structural, and energetic determinants into a common probabilistic framework. PCRPi was upgraded to PCRPi-W^12^ to function as a web server. The users can upload a complex or enter a PDB code and select the type of Bayesian network architecture (expert or naïve) (Assi et al., 2010; Mora et al., 2010). HotPoint^13^ is another hotspot prediction server that uses an empirical formula technique and simple architecture. Its prediction method is based on the contact potential of the interface residues and solvent accessibility developed by Tuncbag et al. (2009). The accuracy of this server is about 70%. The results are a table of interface residues in which the hotspots and their properties are highlighted (Tuncbag et al., 2009, 2010). Grosdidier and Fernández-Recio (2008) have developed an energy-based (Docking) tool with a normalized interface propensity technique. Higa and Tozzi (2009) have developed a tool based on structural and evolutionary methods with the SVM technique. Rajamani et al. (2004) has developed a tool based on sidechain ΔASA (accessible surface area) with the MD technique. HSPred^14^ is an energy-based tool with an SVM (Residue specific) technique (Lise et al., 2009, 2011). MINERVA is a tool based on molecular interaction, structure, and sequence methods with decision tree and SVM techniques (Cho et al., 2009). KFC2^15^ is a server based on various structural features and ASA methods with the SVM technique (Zhu and Mitchell, 2011). Guharoy and Chakrabarti (2009) have developed a tool based on the interface location and H-bonding methods with the simple algorithm technique.

## Peptide–Protein Docking Tools

Molecular docking is a highly applicable method in the design and discovery of small-molecule drugs. This method has also undergone drastic progress in the prediction of PPIs. The prediction of peptide–protein interactions is the subject of similar attempts to develop more amenable peptide therapeutics (Diller et al., 2015; Ciemny et al., 2018). However, docking methods generally struggle with the issue of modeling the considerably more flexible and larger peptide molecules (London et al., 2013). However, peptide therapeutics have recently garnered a lot of attention, which has led to rapid advancements in peptide-protein docking-related technologies. These efforts have resulted in advances in drug design and discovery (Fosgerau and Hoffmann, 2015; Bruzzoni-Giovanelli et al., 2018; Ciemny et al., 2018). There are three main approaches for peptide-protein docking: global docking, local docking, and template-based docking. Different methods have varying degrees of prediction accuracy, often dictated by the amount of interaction information given as input (Ciemny et al., 2018). Template-based docking methods build a model of the complex using known structures, and it can be beneficial if the template is close to the investigated complex (Kundrotas et al., 2012; Lee et al., 2015; Lensink et al., 2017; Marcu et al., 2017; Pallara et al., 2017). Local docking methods look for a peptide-binding pose that is close to a user-defined binding site. Thus, the docking accuracy relies on the input information. The more precise the defined binding site, the better the results (Ciemny et al., 2018). Global docking methods conduct a collaborative scan for the pose and peptide-binding site. The most basic procedure for this approach is considering the protein and peptide input conformations to be rigid and conducting a comprehensive rigid-body docking. Predicting the peptide conformation based on a sequence given by the user is a more complicated approach for this method. Typically, their pipeline includes three phases; primarily, the input peptide conformations should be created by various strategies [e.g., utilizing monomeric protein structure fragments (Yan et al., 2016; Porter et al., 2017), threading the sequence onto a predefined set of template conformations (de Vries et al., 2017), or peptide folding simulation in solution (Ben-Shimon and Niv, 2015)]; then, the docking of rigid bodies should be implemented; ultimately, scoring and, or refinement of the models should be performed (Ciemny et al., 2018). At least three significant challenges lie ahead in the path of efficient peptide-protein docking. The first challenge is the prediction of significant conformational changes in the protein and peptide molecules (flexibility problem) upon docking. The second challenge is selecting the structure with the highest accuracy from a large number of produced models (scoring problem). The third challenge is integrating computational predictions and experimental findings into the peptide-protein docking scheme (integrative modeling) (Ciemny et al., 2018). Table 7 includes features and descriptions of the main peptide-protein docking tools and servers that are currently available.

**TABLE 7:** Peptide-protein docking tools and servers.

Tool or server	URL	Required input	Brief description	References
PIPERFlexPepDock	http://piperfpd.furmanlab.cs.huji.ac.il	N/A	(1) Global docking method, (2) Using Rosetta fragment picker for predicting peptide conformation, (3) Rigid-body docking by using PIPER (Kozakov et al., 2006), (4) scoring by using Rosetta energy function, (5) Rosetta FlexPep Dock for refinement (London et al., 2011)	Alam et al., 2017
ClusPro PeptiDock	https://peptidock.cluspro.org/	N/A	(1) Global docking method, (2) Prediction of peptide conformation based on motif, (3) Rigid-body docking by using PIPER[1], (4) Using structural clustering for scoring	Porter et al., 2017
CABS-dock	http://biocomp.chem.uw.edu.pl/CABSdock and as a standalone version	N/A	(1) Global docking method, (2) scoring based on clustering, (3) Receptor flexibility is typically limited to small backbone, which can be increased if needed	Kurcinski et al., 2015
pepATTRACT	http://bioserv.rpbs.univ-paris-diderot.fr/services/pepATTRACT/	N/A	(1) Global docking method, (2) use ATTRACTscore for scoring, (3) use iATTRACT for flexible refinement of models (Schindler et al., 2015), (4)both interacting residues of receptor and ligand are flexible	Ciemny et al., 2018
Surflex-Dock	Standalone version	Pc and B (binding site region mark by user)	(1) Local docking method, (2) Peptide conformations inside binding pockets are created using a rotamer library, (3) Receptor flexibility is restricted to the pocket of binding site	Jain, 2003
Gold	Standalone version	Pc and B(binding site region mark by user)	(1) Local docking method, (2) Monte-Carlo-based sampling of peptide conformations inside binding site pocket, (3) Flexibility in receptors is either restricted to side chains or is implicit (ensemble docking)	Verdonk et al., 2003
DINC 2.0	http://dinc.kavrakilab.org	Pc and B(binding site region mark by user)	(1) Local docking method, (2) The structure of the receptor remains rigid during docking, (3) for docking long peptides according to AutoDock4, the peptide is divided into increasing length segments.	Antunes et al., 2017
AutoDock Vina	Standalone version	Pc and B(binding site region mark by user)	(1) Local docking method, (2) Monte-Carlo-based sampling of peptide conformations inside binding site pocket, (3) Receptor flexibility is typically limited to side chains, which can be increased to backbone if needed	Rentzsch and Renard, 2015
PEP-FOLD 3	http://bioserv.rpbs.univ-paris-diderot.fr/services/PEP-FOLD3	PcB	(1) Local docking method, (2) sampling of peptide conformations according to Monte-Carlo, (3) clustering of resulting models based on RMSD	Lamiable et al., 2016
HADDOCK peptide docking	http://milou.science.uu.nl/services/HADDOCK2.2/haddock.php	PcB (binding site residues list by user)	(1) Local docking method, (2) Threading a peptide sequence into three peptide conformations results in the creation of peptide structures, (3) rigid-body docking of peptide inside the binding site pocket, (4) binding free energy used for scoring, (5) binding site residues and peptide are fully flexible	Trellet et al., 2013
PepCrawler	http://bioinfo3d.cs.tau.ac.il/PepCrawler/	PcB	(1) Local docking method, (2) peptide is fully flexible and Rapidly exploring Random Trees algorithm using for its docking, (3) use clustering for scoring, (4) the flexibility of Receptor restricted to sidechains	Donsky and Wolfson, 2011
Rosetta FlexPepDock	http://flexpepdock.furmanlab.cs.huji.ac.il and standalone version	PcB	(1) Local docking method, (2) optimization flexible peptide inside receptor pocket based on Monte Carlo, (3) Receptor flexibility is typically limited to side chains, which can be increased if needed, (4)Using Rosetta energy function for scoring	London et al., 2011
PepComposer	http://biocomputing.it/pepcomposer/webserver	B (does not require peptide sequence)	(1) Template-based docking method, (2) In the database of experimentally solved monomeric proteins, look for regions that are structurally close to the region of a predefined binding site	Obarska-Kosinska et al., 2016
GalaxyPepDock	http://galaxy.seoklab.org/pepdock and a standalone version	N/A	(1) Template-based docking method, (2) Look for templates that are identical in form and interaction, (3) energy-based optimization use for model building, (4)scoring is done according to energy	Lee et al., 2015

## Virtual Screening Methods for Peptide–Protein Interactions

Virtual screening (VS) is a robust computational technique capable of searching huge libraries of small molecules and identifying the most suitable ones against a protein receptor. VS has emerged as a complementary technique of high-throughput screening (HTS). Nowadays, thanks to the advancements in VS, it has become an indispensable part of the drug discovery process, leading to enormous savings in cost and time. Recent studies have established that VS can help to develop inhibitors of PPIs.

Exploration of new macromolecular structures by NMR or x-ray crystallography methods, human genome sequencing, and rapid computational methods could improve VS searches. Although increasing the numbers of the unveiled structures of protein–ligand complexes has made VS more convenient, 3D structure prediction approaches could be employed where experimentally determined structures of proteins/peptides are not available (Slater and Kontoyianni, 2019).

There are several accessible public databases for known drugs, small molecules, and chemical compounds (natural or synthetic) which could be searched when implementing VS strategies: ChemDB^16^, ChemBank^17^, NCI Open Database^18^, ChEMBL^19^, PubChem^20^, ZINK^21^, ChemSpider^22^, and DrugBank^23^ (Lavecchia and Di Giovanni, 2013). There are also some commercial databases containing data derived from patents or literature, including ACD^24^ and WOMBAT^25^ (Lavecchia and Di Giovanni, 2013). There are two main categories of VS: ligand-based VS (LBVS) and structure-based VS (SBVS).

### Ligand-Based VS

The LBVS approach relies on the extracted structural and bioactivity information from an enormous small-molecule library. Three-dimensional shape matching is one of the popular LBVS methods based on seeking molecules with a similar shape to that of the known active molecules. Utilizing pharmacophore models to find the intended ligand, quantitative structure–activity relationships (QSAR), and chemical similarity analysis (to look in a database of molecules against one or more active ligand structures) are additional LBVS methods.

Similarity analysis is one of the most common techniques of VS. In the similarity searching method, the closest molecules are identified for a known active reference structure. Nevertheless, this method is simply influenced by the users because the selection of accurate input molecules is a challenging issue. However, this is a fast VS method (Lavecchia and Di Giovanni, 2013; Wu et al., 2019).

Pharmacophore modeling is another method for LBVS. A pharmacophore is a set of crucial molecular properties involved in accurate molecular recognition and interactions of a ligand with a specific biological target. Since 3D models are necessary for docking, pharmacophores could serve as vital VS processes to explore novel ligands for receptors with unknown 3D structures. There are various pharmacophore-based VS case studies for peptides. For example, a study designing the pharmacophore model for secretin resulted in gaining novel angiotensin-converting enzyme (ACE) inhibitory peptides with the desired biological activity. Angiotensin-I-converting enzyme inhibitory peptides and Intestinal Peptide Transporter hPepT1 are some of the more successful examples of pharmacophore modeling in LBVS methods (Dong et al., 2011; Wang et al., 2011; Osmulski et al., 2020). However, pharmacophore modeling has some limitations, especially when working with peptides. Some of the snags present in peptide-based pharmacophore modeling are the lack of acceptable scoring metrics, lack of clear instructor pharmacophore query, improper binding affinity evaluation, and incorrect or inadequate conformational sampling. These limitations could lead to false-positive and false-negative results (Kaserer et al., 2015; Ciemny et al., 2018; Slater and Kontoyianni, 2019).

Quantitative structure–activity relationship is one of the most popular LBVS methods. This technique could involve the interplay between biological function, the potency of active molecules, and their structural/physicochemical features. QSAR-based methods need information such as the values of IC_50_ and the binding affinity (*K*_*d*_). QSAR modeling is classified into 2D-QSAR and 3D-QSAR; 2D-based algorithms are faster but less precise in comparison with 3D-based algorithms. The solubility, flexibility, ligand and protein conformations, and structures are not considered in both of the methods (Kanakaveti et al., 2020).

### Structure-Based VS

The SBVS methods require knowledge of the 3D structures of proteins and not the biological function of known molecules. SBVS involves docking the candidate molecules with the protein target, followed by ranking the predicted binding affinity by scoring functions to detect potential lead candidates. In this approach, the 3D structure of the protein of interest should be available through x-ray crystallography, NMR, or homology modeling. Subsequently, docking could engage the small molecules as ligands of the receptor *via* computational algorithms; then, the top-ranked compounds could be selected for further experimental studies. Scoring of the ligands through different scoring functions (empirical, knowledge-based, and force field-based) is a critical step in SBVS. The flexibility of the target structure is another complicated aspect of SBVS, which could noticeably compromise the accuracy of the approach. In recent years, docking algorithms are seriously confronting this challenge by improving soft docking. MD simulation, which considers ligand flexibility and entropic effects, could also be combined with SBVS or LBVS to increase the information accuracy of the binding pose of candidate molecules and subsequently improve the drug development (Yan et al., 2019; Wang and Sun, 2020).

### Combining Ligand-Based and Structure-Based Approaches

The drawbacks and limitations of traditional VS methods could be alleviated by combining LBVS and SBVS. This unified approach applies both structural similarity and ligand-based data. A researcher could select one of the LBVS, SBVS, or integrated approaches depending on the case study. One of the successful reports in recent years is the designing of the polo-box domain of polo-like kinase 1 (PLK1-PBD) inhibitor through a combined strategy of SBVS pharmacophore modeling and molecular-docking screening techniques (Ramezani et al., 2019; Yan et al., 2019). The study resulted in the discovery of a peptide as a potent candidate for further experimental investigation. Notwithstanding the successful results gained by the combined approach, critical improvements in the combination strategies are still needed. One of the fundamental limitations is the complicated procedure of the platform handling, so improving the performance of this process would bring new advancements soon (Wang and Sun, 2020).

## Optimizing Interfering Effects of Peptides

The interfering effects of peptides could not be accessed accurately by biophysical techniques such as x-ray crystallography, NMR spectroscopy, and fluorescence spectroscopy. They have unique features such as high selectivity and immunogenicity (Bruzzoni-Giovanelli et al., 2018). Despite their advantageous features, they could exhibit limited cellular penetrability, low *in vivo* stability, low solubility, and low binding strength. However, various methods have been considered to improve these limitations. The peptides could be optimized by chemical, biophysical, and *in silico* methods (Bruzzoni-Giovanelli et al., 2018). *In silico* methods of peptide optimization seem appealing due to their computational nature, which have the advantages of low cost, less time consumption, and avoiding the ethical issues of empirical analyses. There are some modifications that can be made to the *in silico* methods which can improve the efficacy of interfering peptides.

One way to optimize the performance of interfering peptides is the utilization of peptide design tools. Peptide design tools predict the spatial and energy constraints applied to them and suggest the best folding. Two main peptide-designing approaches include the stochastical and deterministic methods. In the deterministic approach, a complete space sequence is searched to reach the sequence fold with the lowest formation energy. In contrast, the stochastical method searches for the sequence space heuristically, which includes MC and genetic algorithms. The structure of the interfering peptide is fixed by optimizing the rotation angles of the lateral chains and energy minimization is performed by tools such as Rosetta Design. This software also improves the network of hydrogen bonds and van der Waals interactions (Roy et al., 2017). It should be noted that the scaffold can be changed during the modification of amino acids. Most methods such as GRAFTER, FITSIT, Proda Match, and Scaffold selecting suitable peptide patterns require geometric constraints such as the coordinates of the pattern-representing atoms. The AUTO match tool exhibits the flexibility of backbone patterns. When a correct peptide pattern is chosen, side-chain amino acids need to be modified to improve their correctness. Side chain changes can be evaluated by various factors, such as determining the binding affinity and structural stability. Tools such as ORBIT are used to reform the side chain that can bind to the target molecule (Roy et al., 2017). PPIs have a crucial role in signal transduction processes. A rapid computational approach has been developed to predict energetically critical amino acid residues in PPIs. The input consists of a 3D structure of the protein–protein complex. The output is a list of “hot-spot” or amino acids side chains that are predicted to become unstable when mutated to alanine (Kortemme et al., 2004). *In silico* alanine scanning could be used to improve the efficacy of the interfering peptides.

Poor cell membrane permeability of interfering peptides is a significant hurdle. Therefore, improvement of membrane permeability or development of strategies that facilitate active intracellular uptake will be critical for successful peptide-based targeting of intracellular PPIs. Increased uptake could be achieved by the identification of unnecessary hydrophobic amino acids. These amino acids can be replaced by charged or polar residues while maintaining their native bioactivity. Recently, *in silico* methods have been applied to speed up this process (Lee et al., 2019). CcSOL Omics is software that is used for the prediction of proteome solubility and the identification of solvent motifs according to the given amino acid sequence. PROSO-II is another SVM-based tool that predicts the solubility of a peptide based on physicochemical properties such as hydrophobicity, hydrophilicity, and features of secondary structure (Lee et al., 2019).

One of the main impediments ahead of clinical development of interfering peptides is their high sensitivity to proteases. Their half-life is highly reduced by proteolytic cleavage. The most common structural changes that increase protein stability are chemical changes. These optimizations include the acetylation of N-terminus and C-terminus ends of the peptide scaffold, the introduction of dextrorotary (D)-amino acids, and peptide scaffold cyclization (Sorolla et al., 2020). Engineering peptides by introducing D-amino acids instead of levorotatory (L) forms is an effective strategy to avoid proteolytic degradation by proteases. These D-amino acids could cause structural changes in the target peptides, which makes them unrecognizable by proteases. Recent studies have shown that interfering peptides containing D-amino acids have a longer half-life. Many therapeutic peptides containing unnatural amino acids have been approved by the FDA, such as degarelix for prostate cancer, semaglutide for type 2 diabetes, and carbetocin (an oxytocin analog containing methyl-tyrosine) for postpartum hemorrhage (Sorolla et al., 2020). Cyclization of peptides can increase their half-lives. This cyclization can be accomplished by different methods, such as the formation of a disulfide bond between cytosine, adding an amide bond between the C- and N-terminus (head to tail), and addition of amide bond between natural amino acid side chains (side chain cyclization). Click chemistry software is a widely used tool for designing cyclic peptides. PEP-Cyclizer is another software that is used to design head-to-tail cyclization. This software has other complementary features such as the ability to search for candidate sequences compatible with the cyclization of the peptide (a facility to assist medicinal chemists), the generation of 3D models of a cyclic peptide starting from the 3D structure of the un-cyclized peptide and the sequence of the cyclized peptide, and preliminary steps for the conformational stability analysis of other peptides, or peptide-receptor docking (Pierce et al., 2014).

## Peptide–Protein Binding Energy Calculation Tools

The value of determining the binding energy of protein–ligand interactions in docking studies is apparent in the field of drug design. Acquisition of sufficient knowledge at a molecular level leads to accurate simulation, a valuable tool for drug design purposes. Experimentally measuring an inhibition constant (Ki) of the enzyme or protein in the presence of both the inhibitor and the substrate can estimate the binding affinity. In theoretical calculations, evaluating the properties of the protein, ligand, and after that, the complex can estimate the free energy of the ligand. Theoretically calculated free energies of the binding are often compared with the free energy of the reaction by calculating the *K*_*d*_. Molecular mechanic force fields, which play a vital role in the conformational flexibility studies, like AMBER, MMFF, CHARM, and OPLS, can calculate various interactions in non-bonded atoms (Vanommeslaeghe et al., 2014; Ciemny et al., 2018). There are several methods for calculating the binding energy, namely endpoint, pathway, and alchemical methods.

### Endpoint Methods

Endpoint methods are fast; however, their accuracy is usually limited to rational ranking (with r2 starting from 0.4 to 0.9, with a median of ∼0.7). The main advantage of endpoint methods is the requirement of only the simulation of the bound and free states of the ligand compared with pathway methods, which demand the simulation of many intermediate states likewise. Molecular mechanics/generalized Born surface area (MM/GBSA) and molecular mechanics/Poisson–Boltzmann surface area (MM/PBSA) are two general similar endpoint methods employed in protein–ligand-free energy calculations (Wang et al., 2019). Changes in solvation free energy, molecular mechanical energy, and conformational entropy are utilized to calculate the free energy of binding (Hall et al., 2020).

Linear interaction energy (LIE) is another endpoint approach used to figure out binding affinities. LIE relies on the concept that the free energy of binding shows a linear dependency on the polar and non-polar variations in ligand-surrounding energies. The strategy involves calculating values for the protein–ligand binding free energy ΔG bind with conformational sampling. Efforts such as considering multiple binding poses and continuum electrostatics solvation have promoted the accuracy of the LIE method (Rifai et al., 2020).

### Chemical Methods

Although physically non-realizable, there are feasible computational processes of moving a set of atoms in a system from one state to another. In such a situation, modeling by alchemical free energy calculations could be one of the helpful computational processes. In strategies based on alchemical modifications, shifting the ligand into non-interacting mock particles may offer the absolute free energies of binding. Further, calculating the difference of free energy between two ligands can test the relative free energies of binding (de Ruiter and Oostenbrink, 2011). Free energy perturbation (FEP), Bennet’s acceptance ratio (BAR), and thermodynamic integration (TI) are typical samples of alchemical methods, in which the statistical mechanics provide the demanded data. Performing more extended simulations may yield way more accurate energy calculations (Ciemny et al., 2018).

In addition to established methods, some prefer using combinations of existing approaches or try novel strategies. RETI is a successful combination of TI and replica exchange (RE). TI is known as a powerful method used to calculate free energy differences. However, when several confirmations are involved, getting accurate outcomes becomes problematic. RETI can somewhat solve the problem by enhancing sampling efficiency. Enhanced sampling-one-step perturbation (ES-OS) and linear interaction energy/one-step perturbation (LIE/OS) are among other combined approaches. The aim of using the LIE/OS method is to enhance the accuracy of energy calculation. LIE calculates the charging atoms in the interaction cavity, while OS is applied to explain the contributions from cavity formation (de Ruiter and Oostenbrink, 2011).

### Path Sampling

Path sampling approaches can meet the computational aspect of a process using the separation of timescales in biomolecular systems. Thus, it is advantageous when used in process of non-clear separation timescales. Path sampling methods compute functional transition rather than stable states, by physically removing the ligand from the protein and calculating the mean force along this path. Similarly, path sampling can be used efficiently for the conformational sampling of stable states fragmented by low barriers. Transition path sampling (TPS) and MC simulations are two approaches using a complete path. TPS is helpful for chemical or physical transitions of a system from one stable state to another state (such as protein folding or chemical reactions) that infrequently occur to be observed on a computer timescale (Chong et al., 2017).

There are some vital factors to be considered in using these methods, which might remarkably affect the accuracy and computational time of calculation. Deciding on the solvent, the simulation length, and ligand orientations, following the selection of relative or absolute free binding energies, is a critical step in the calculation process (de Beer, 2012).

### Common Issues

Improving the accuracy and the speed of calculations simultaneously is the main aim of the methods described above; however, it seems challenging. As an example, factoring in solvent interactions will enhance the accuracy but also will increase the required computational time. Including explicit water molecules in the binding sites will increase the accuracy for specific systems. In explicit water simulations, grand canonical MC simulations (GCMC) can determine the fluctuation of the number of water molecules in the binding site. Paying attention to polarizability, accurate force field, and charge transfer is crucial in exact free energy calculations; their effects can be considered using quantum mechanics. Quantum mechanics can efficiently calculate electrostatic interaction energies. However, it would not be affordable in the study of massive molecules. Semi-empirical quantum methods are essential in computational chemistry for treating large molecules and studying electrostatic interactions in peptide-protein interactions. Semi-empirical approaches are generally based on the Hartree–Fock formalism with some more approximations and empirical data usage (de Beer, 2012; Rifai et al., 2020).

As mentioned above, a combination of the present methods has been recently developed to overcome the sampling problems and other shortcomings of the methods on their own, while strengthening their advantages. It is assumed that these combined methods would result in more accurate energy binding calculations, consequently having more reliable outcomes for application in drug design developments.

## Discussion

*In silico* methods are implanted into every corner of the biological analyses. The study of IPs capable of modulating the PPIs is no exception and neither are the extended *in silico* tools established to develop, analyze, and optimize the IPs. These methods are anticipated to grow and become more accurate by reiterating the cycles of prediction and empirical assessment of these predictions. More precise algorithms would be developed, including the parameters of new players discovered by empirical studies. The novel and more accurate *in silico* tools would design and optimize an almost unlimited number of IPs with a high affinity against extracellular or intracellular (CPPs) PPIs. These tools would also help develop pharmacokinetically stable, safe, and effective IPs to modulate the PPIs within the cellular membranes. Moreover, these tools would be employed to predict, exert, and analyze specific chemical modifications to improve the ADME properties of the IPs. They also would be harnessed to design multifunctional IPs and even remotely controlled PPI inhibitors. Moreover, available natural peptides, evolutionarily selected for high stability and specificity, would be employed by *in silico* tools for rational structure-based design of IPs harboring similar properties. The ever-growing need for more effective therapeutics would be the driving force for further growth of the *in silico* methods in IP development. Given the apparent advantages of the peptides over small molecule drugs, higher investment of the pharmaceutical industry into the development of IPs does not seem farfetched. *In silico* tools will play a pivotal role in the transition from small molecule-based modulators of PPIs to peptide-based modulators.

Modulation of PPIs using the IPs is one of the hot research topics of various scientific fields such as biochemistry, chemical biology, and pharmacology. Recent progress in developing *in silico* methods for the evaluation and identification of these IPs has hugely extended the field. Although some issues about the pharmacodynamics and pharmacokinetics of peptides remain to be addressed, many companies have already devoted themselves to peptide discovery, which resulted in numerous peptide drugs and homologous compounds on the market. *In silico* methods could offer a comprehensive and exciting portfolio of applications in IPs targeting the PPIs. The interest in these methods is reflected in the increasing number of algorithms, software, and related publications. These methods have a lot to offer toward resolving challenges like low membrane permeability, the tendency for aggregation, short half-life, fast elimination (if not stabilized), proteolytic degradation, specific targeting, toxicity, immunogenicity, and optimization of IPs. Each issue could be dealt with by using a combination of *in silico* tools developed based on the experimental results. Most of the peptide-related challenges require atomic-level structural information. The structural information about the peptides could be found in structural databases. The *in silico* methods could predict the structure of peptides lacking the previously resolved 3D structures. Given the 3D structure of an IP, its properties could be analyzed and optimized to circumvent functional limitations. These structures could also be employed to screen peptide libraries and find new targeting peptides. The efficacy of *in silico* tools highly depends on the accuracy of their algorithms. These algorithms are continuously revised considering the evidence obtained from experimental studies. Given their high potential to unravel the questions about IP design and optimization, *in silico* tools would become an inevitable part of the development of IPs that modulate the PPIs.

## Author Contributions

SK, AJ, and MR contributed to the conception and design of the study. All authors contributed to the study data collection and data analysis, wrote the first draft of the manuscript, commented on previous versions of the manuscript, and read and approved the final manuscript.

## Conflict of Interest

The authors declare that the research was conducted in the absence of any commercial or financial relationships that could be construed as a potential conflict of interest.

## Footnotes

1 www.neuropeptides.nl

2 http://www.uniprot.org

3 https://www.rcsb.org

4 http://www.imtech.res.in/raghava/pepstr/

5 http://www.bioinfo.rpi.edu/~bystrc/hmmstr/about.html

6 http://pepsite2.russelllab.org

7 http://flexpepdock.furmanlab.cs.huji.ac.il/

8 http://www.eppic-web.org/ewui/

9 http://foldxsuite.crg.eu/

10 https://robetta.bakerlab.org/

11 http://ftmap.bu.edu/serverhelp.php

12 http://www.bioinsilico.org/PCRPi/

13 http://prism.ccbb.ku.edu.tr/hotpoint/

14 http://bioinf.cs.ucl.ac.uk/psipred/

15 https://mitchell-web.ornl.gov/KFC_Server/index.php

16 http://cdb.ics.uci.edu

17 http://chembank.broadinstitute.org

18 http://cactus.nci.nih.gov/ncidb2.2/

19 https://www.ebi.ac.uk/chembl/

20 http://pubchem.ncbi.nlm.nih.gov

21 http://zinc.docking.org

22 http://www.chemspider.com

23 http://www.drugbank.ca

24 http://accelrys.com/products/databases/sourcing/avaible-chemicalsdirectory.html

25 https://www.proofpoint.com/us/wombat-security-is-now-proofpoint
